# Resilient rural women’s livelihoods for poverty alleviation and economic empowerment in semi-arid regions of Zimbabwe

**DOI:** 10.4102/jamba.v10i1.524

**Published:** 2018-09-12

**Authors:** Hilda Jaka, Elvin Shava

**Affiliations:** 1 Department of Public Management and Development, North-West University, South Africa

## Abstract

The purpose of this article was to examine the implementation of rural women’s livelihoods towards economic empowerment of women in Chivi District of Zimbabwe. A drought-ridden and semi-arid district because of climate change, Chivi District receives very low annual rainfall which impacts negatively on agriculture as the main rural women’s livelihood among others such as craftwork, pottery, gardening and poultry projects. Using a case study approach that triangulates interviews, focus groups and documents, the article found out that women faced numerous challenges. The findings of the study revealed that in their quest to reduce poverty and economically empower themselves, women encountered the lack of access to competitive markets, micro-insure rural women’s livelihoods, lack of access to credit facilities, lack of entrepreneurial education and training, effects of climate change, limited use of technology to stimulate rural women’s livelihoods. The article concludes that to achieve economic empowerment through resilient rural women’s livelihoods, access to competitive markets and entrepreneurial education supported by adequate funding is fundamental.

## Introduction

Rural women around the world are instrumental in poverty reduction and sustainable development in communities and households through a myriad of livelihood strategies (Fonchingong 2005; Mutopo 2014). In developing countries, women make important contributions to the rural economies through livelihoods, as farm labourers on their farms or as wage labourers on other people’s farms, producing for their own consumption or for selling or both (Fonchingong 2005; Mutopo 2014; Scoones 2009; Vercillo 2016). Although their roles vary a great deal in different regions, they manage complex households and are involved in multiple livelihood strategies. FAO IFAD and ILO (2010) state that women’s roles include agricultural work such as livestock production, food processing and preparation, a collection of firewood and water for household use and home maintenance. These activities are not included as ‘economically active employment’ in national accounts but are imperative to their household’s well-being (SOFA Team & Cheryl 2011). Although these livelihood activities require women to work for long hours, women’s efforts are negatively affected by socio-economic inequalities and marginalisation (Zaidi & Munir 2014).

Poverty reduction and sustainable development rely mostly on empowering people, especially the poor people. One of the avenues to empower the poor and rural women is through interventions for sustainable livelihoods. Women play a key role in rural livelihoods for household food security and income generation (Kristjanson et al. 2014; McKee 1989; Mutopo 2014; Oberhauser, Mandel & Hapke 2004). Livelihoods are termed as the activities and the resources done to gain a living. The most common definition of livelihoods encompasses the capabilities, assets and activities used in order to gain a living. Central to the sustainability of livelihoods is the livelihood assets, which are the means of production available to communities to generate material resources in order to survive (Vercillo 2016). Sustainable livelihoods are those that can cope with and recover from vulnerabilities. These should be able to maintain their form and structure, maintain their capabilities and assets presently and in future without negatively affecting the natural resource base. The process of sustainability relies hugely on the resilience of rural livelihoods to various challenges for the empowerment of rural women (Davies et al. 2013). The multiple and diverse constraints include the lack of appropriate technologies, unequal access to productive resources and services, inadequate or inaccessible infrastructure, limited access to credit, health care and education, global food and economic crises and climate change (Garikipati 2010). In sub-Saharan Africa, rural women rely on agriculture as their main source of food and other diverse livelihoods as their source of income (Mutopo 2014). Therefore, any initiative that empowers them to reach their full capacity will not only empower women but also drive the socio-economic growth of communities (Ellis & Freeman 2004; Frost et al. 2007; Kristjanson et al. 2014; Scoones et al. 1996).

Hill (2011) opines that rural women’s livelihoods in semi-arid regions can contribute towards poverty reduction and women’s economic empowerment as women become independent to generate their own income for sustaining their livelihoods. The Government of Sweden (2010) admits that women’s economic empowerment increases women’s access to opportunities and resources, which include jobs, properties, assets and financial services. Therefore, this article argues that women’s economic empowerment through livelihood development in semi-arid regions is tantamount to repositioning women and improving the well-being of their families (OECD 2010). To this end, therefore, there is a need to motivate for the creation of resilient livelihood strategies and much more resilient communities for survival. Diverse rural livelihoods can be used, for rural women, to bridge gender disparities, discrimination and ethnicity challenges, as well as reducing poverty, and act as coping strategies to disastrous shocks and stresses in semi-arid regions where economic opportunities are limited (Maroyi 2009; Shackleton, Shackleton & Cousins 2001). Livelihoods of people cannot be sustainable if they are not resilient. A livelihood is sustainable when it can cope with all the shocks and stresses and recover unchanged; it should be able to enhance capabilities and assets without undermining the natural resource base (Gwimbi 2009).

This study used the sustainable livelihoods theory to explore the sustainability of rural women’s livelihoods in the semi-arid regions of Zimbabwe, in particular, Chivi District. FAO (2011) claims that, although livelihoods diversification has been highly supported for rural households, agriculture has remained the main livelihood for rural women to promote food security, even in drought-prone regions. Gangata and Matavire (2013) assert that rural areas in Zimbabwe have suffered various shocks and stresses; these include persistent economic meltdowns and climate changes, such as droughts and floods that negatively affect agriculture. These shocks have left a wake of poverty, hunger and a general decline in living standards, especially for people in the rural areas with limited access to resources. These drastic changes have also facilitated shifts for women to play active roles in income-generating activities (Fook 2011).

## Statement of the problem

Various disasters and challenges have an impact on livelihoods of rural women in Zimbabwe (Scoones 2009). The effects of climate change present devastating consequences on many districts in Zimbabwe, of which Chivi District is no exception. Chivi District normally receives low annual rainfall as it lies in a semi-arid region, which has threatened rural women’s livelihoods. The increasing poverty because of various challenges forced some women to embark on numerous livelihood strategies that include pottery, gardening, poultry farming, community-driven irrigation farming, sewing and credit clubs, to generate employment and improve household income. However, these rural women’s livelihoods are not sustainable due to the lack of financial, physical and human capital; and lack of social, natural capital and household assets that enable viable livelihood execution. Several studies have shown that agriculture as the main rural women’s livelihood is being constrained by dry spells, flooding, cyclones, droughts and heat waves which affect crop production (Gwimbi 2009; Matondi 2011; Scoones 1996; Scoones et al. 1996). This article, therefore, observes that rural women’s livelihoods are not resilient and this may be because they are not being adequately supported and funded by either government or stakeholders. There is a need to create and boost resilient livelihoods for rural women to reduce poverty and promote women’s economic empowerment (Davies et al. 2013). The article, therefore, adopted a sustainable livelihood approach to assess the resilience and sustainability of livelihoods of women in Chivi District.

## Aim and objective

The main aim of this study was to explore the livelihoods of rural women for poverty alleviation and economic empowerment in the semi-arid region of Chivi District in Zimbabwe. The main objective of this article was to explore different strategies that can be used to empower women through livelihoods in Chivi District. The article stresses the importance of empowering rural women as transformation agents for increasing household income in semi-arid regions. The article sought to close this gap by answering the following questions: Do rural women’s livelihoods mitigate poverty and improve household income in Chivi. Are rural women’s livelihoods resilient to various stresses to promote women’s economic empowerment in semi-arid regions of Zimbabwe?

## Literature review

In the quest to gain familiarity with the livelihoods of rural women and the challenges faced, an extensive review of the literature was done. This section with varied sub-topics pursued an understanding of rural women’s livelihoods and the challenges they face. Theoretical explanations were also explored to understand the phenomenon. The literature review was done using the following sub-topics to promote clarity: the global context of rural women’s livelihoods, resilient pathways in semi-arid regions and rural women’s livelihoods in semi-arid regions of Zimbabwe. The sub-topics were used to promote focus and allow for an in-depth analysis of concepts.

### The global context of rural women’s livelihoods

Livelihoods are the strategies that people perform to satisfy their needs and earn a living (Bryceson 2002). These livelihoods are possible activities to earn an income and could be from hired employment, self-employment, remittances or a combination (Gwimbi 2009; Kristjanson et al. 2014; Mutopo 2014; Shackleton et al. 2001). Sustainable livelihoods are those livelihoods that are sufficient enough to avoid poverty and improve the general well-being of households and families (FAO 2011). Rural livelihoods imply the systems in place for rural people to make a living, whether their livelihoods are secure or vulnerable over time. Livelihood security is the secure access, availability and ownership of resources, assets and reserves to ease shocks meet contingencies and offset risk (Gerbens-Leenes & Nonhebel 2005; Gladwin et al. 2001; Hermann et al. 2012).

Lin (2011) and Mudimu (2002) argue that rural women globally encounter various issues that include the changes occurring because of globalisation and technological advancement, policies and external interventions, access to and control over resources, women in leadership and strengthening capabilities such as education, health and equality. Although agriculture has remained the major employer of rural women across the world, especially in sub-Saharan Africa and Southern Asia, it has emerged to be a challenge to sustain households based on agricultural activities alone (Batterbury 2008; Bryceson 2002; Davies et al. 2013; Ejigu 2008; FAO et al. 2010; Zeitoun 2011). In an attempt to achieve economic empowerment, rural women have adopted numerous livelihood strategies to supplement their income they obtain from agriculture. Nevertheless, this diversification is understood to be controlled by factors such as access to resources and education (FAO et al. 2010; Mutopo 2014; O’Laughlin 1998; UNDAW & UNIFEM 2001). The diversification may also take different forms depending on the country. In Latin America, women work for bigger agribusiness companies, for example, the flower plantations of Ecuador, and earn wages seasonally. In Thailand, women have sub-contracted their family plots for the production of baby corn and asparagus for a bigger company (UNDAW & UNDESA 2008). Some women raised shrimp under contract with big companies for export, and this has seen women earning more than they did when they grew rice and also has cut down the hours women have to be working (UNDAW & UNDESA 2008). In South and South-East Asia, many rural women were forced to migrate to urban areas to look for employment opportunities because agriculture was failing. In Bangladesh, the larger number of rural women migrating for work opportunities in urban areas were unmarried and divorced, with only basic levels of education (Davies et al. 2013; UNDAW & UNDESA 2008). These women would find themselves working in textile industries, which employ many women (UNDAW & UNDESA 2008). Oberhauser et al. (2004) argue that rural women use a myriad of livelihoods in order to meet their responsibilities. For instance, women in Porto Novo were said to be indulging in selling food as it brought regular income and created an avenue for food availability for their families (Oberhauser et al. 2004). In developing countries, studies have shown that despite constraints and challenges they face, women still reserve the right to choose a form of livelihood strategy which befits them, and this has provided a sense of ownership guaranteeing hard work and dedication from the women (UNDAW & UNIFEM 2001). In countries such as Pennsylvania and South Africa, this freedom of choice has enabled women to hold relative decision-making powers on what is best for their families (Oberhauser et al. 2004). Nonetheless, decisions are affected by the availability and accessibility of markets, infrastructure, religions and customs that prohibit women’s participation in economic activities. For countries in semi-arid regions, such as Zimbabwe, South Africa and Botswana, the choice of livelihoods is affected by various challenges; some of these are natural such as droughts, cyclones and other aspects that are detrimental to the execution of livelihood strategies (Campbell et al. 2002; Ellis 2000; Frost et al. 2007). In such situations, rural communities find ways of coping with such scenarios and their ability to deal with such shocks culminates to community empowerment (Kabeer & Natali 2013). Resilient communities use ways to manage risks that promote sustainable development and enable transformation.

### Resilience pathways in semi-arid regions

The context that makes a sustainable livelihoods theory relevant to resilience building empowerment of women and reduction of poverty is the increased exposure to risks and vulnerabilities faced by rural women on livelihoods (Davies et al. 2013), which in turn affects negatively on family well-being. This article focuses on building resilient livelihoods in order to empower rural women. Resilient livelihoods are those that are protected from shocks and stresses to make community systems more resilient and capable of cushioning the impacts and recover from disrupting events (Batterbury 2008; Charman 2008; Chazovachii & Chuma 2013; Scoones 2009). Resilience refers to the capacity to return to equilibrium after a disturbance, or the amount of disturbance a system can handle before the change of state (Holling 1973; Klein, Nicholls & Thomalla 2003; Maleksaeidi & Karami 2013; Neubert et al. 2011). In a study by Nelson and Stathers (2009), resilience in the socio-ecological context refers to the process of using resources, abilities and adaptation capacities to cushion shocks and stresses while ensuring self-organisation and enabling recovery. Birkmann (2006) and Davies et al. (2013) posited that resilient livelihoods are able to cope with and recover from challenges, shocks and stresses without undermining the environment, and they should be reliable and sustainable to provide enough for households. Therefore, resilience becomes a process of cushioning shocks and stresses arising by using different resources and capacities to achieve a state of adaptation. A resilient system has the potential to achieve sustainability and security (Klein et al. 2003). Resilience means despite the shocks, stresses and other extreme events, the systems will still have the same identity, same structures and function in the same way (Folke et al. 2002). Resilience also includes learning, coping with events, adaptation as well as recovery from stresses arising ending in the improvement, advanced and sustainable ways of dealing with the ever-changing and unpredictable issues (Perrings 2006; Rockström 2003).

Pathways to sustainable resilient livelihoods call for a number of actions to be considered that promote the empowerment of rural women and household well-being. These interactions should include the improvement of access to resources, improvement of agricultural practices, diversification of livelihoods and the general empowerment of women (Gabrielsson & Ramasar 2013; Mortimore et al. 2009). Rural women in semi-arid regions face challenges in dealing with disasters, shocks and stresses that arise because they lack access to information and coping mechanisms (Davies 2015). Livelihoods in Chivi District depend on crop production, which is highly seasonal; small livestock production like chickens and goats, *maricho* (casual employment) in wealthier households; and, in some cases, gold panning, especially in areas along Runde River (WFP 2014). Resource endowments in this area are poor, and the area is susceptible to extreme natural hazards caused by climatic changes such as droughts, floods and heat waves. Poverty levels are very high and women emerge as the most vulnerable (WFP 2015).

In this article, resilience promotes ways in which rural women deal with challenges arising from their own environments. Resilient livelihoods are important for fostering human well-being, better living standards and accelerating access to basic needs by empowering women economically (Pelling 2011). Formation of resilient livelihoods draws on the remembrance of ways and strategies, which have been used over time to try to cope with challenges affecting the livelihoods of rural women (Lin 2011; Maleksaeidi & Karami 2013; Nelson & Stathers 2009; Neubert et al. 2011). Some authors argue that there is no sustainability without resilience and that any development strategy formulated will not be sustainable if it is not resilient (Klein et al. 2003; Perrings 2006). The social aspect of resilience was added as scholars acknowledged the importance of humans with their ability to visualise, foresee, predict and plan which enhances the resilience of a system (Perrings 2006). Therefore, resilient livelihoods may also enable communities to survive disasters and poverty, and achieve sustainability. The argument is that to attain sustainable livelihoods, farmers should be inventive and resilient (Maleksaeidi & Karami 2013). They should have the capacity to withstand shocks and stresses that occur with climate change and the environmental processes; they should be able to improve, withstand and recover (Maleksaeidi & Karami 2013). The review revealed the importance of promoting resilience building through livelihoods for the empowerment of rural women and households. The importance of empowering women is imperative in ensuring that rural women have access to resources that are needed by their households. As alluded in previous literature, the importance of rural women in development and the roles played by women in societies will be more defined with more positive effects if they have resilient livelihoods.

### Rural women’s livelihoods in semi-arid regions of Zimbabwe

Zimbabwe has an agro-based economy that provides the largest avenue for employment and income generation, food for families, as well as a source of direct nutritional food in most households (Chirau, Nkambule & Mupambwa 2014; Makura-Paradza 2010; Maroyi 2009; Matondi 2011; Mutopo 2014; Vercillo 2016). Agriculture tends to receive more attention from people, organisations and the government in terms of funding, subsidies and research. According to ZimVAC (2017), about 80% of the population in Zimbabwe relies on agriculture. In another study by Matondi (2011), rural populations in Zimbabwe attempt to secure their livelihoods by focusing on both crop production and animal production. In most areas of rural Zimbabwe, production of crops such as maize, cotton, tobacco, small grains, soya beans and sunflowers has become a seasonal occupation leading to the adoption of other livelihoods off-season (Bird & Shepher, 2003; Makura-Paradza 2010; Ncube 2012). In other studies by Matondi (2011) and Mutami and Chazovachii (2012), it was noted that market gardening, pottery, traditional beer brewing, sewing and crocheting as well as buying and selling of small commodities have provided for extra income needed during off-seasons and extra occupations in a season.

## Description of the study area

**FIGURE 1:**
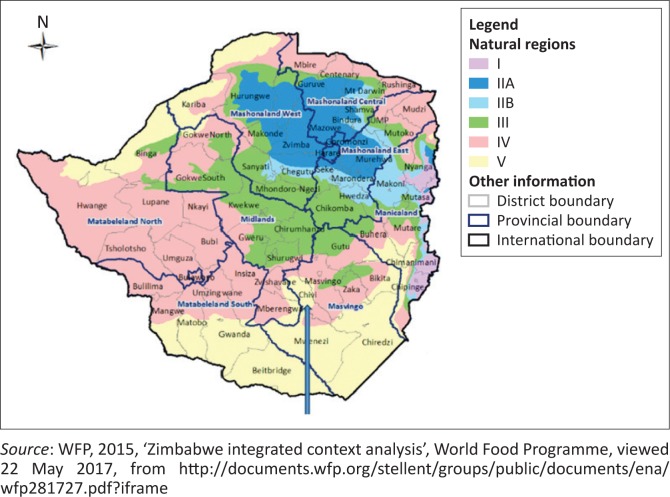
Map for Chivi District in Zimbabwe. Arrow showing Chivi District in Zimbabwe.

Chivi District is situated 60 km – 70 km southwest of Masvingo town in Zimbabwe. The area lies within the agro-ecological regions four and five, which are semi-arid regions highly characterised by poor rainfalls, high temperatures and continuous dry spells (Gerhardt & Nemarundwe 2006; Scoones et al. 1996). Poor soils in this area cannot produce good yields without the use of fertilisers or manure, which are not readily available to all households. The rainfall that is received per annum is 530 mm on average, and drought is a recurrent phenomenon occurring almost three years out of every five years (Gerhardt & Nemarundwe 2006). The pressure on land is increasing with the rapid population growth rate. The surveyed villages are Jenya village, which falls in Ward 7; Zhara village which falls in Ward 20; and Hapazari and Charashika villages, which fall in Ward 14. According to the census data, the district has a recorded population of 166 049 people, with 54.3% females and 45.7% males. The total 97.4% of these people live in the rural area, while 2.6% live in urban with a density of about 70 people/km^2^ (Zimstats 2012). Individual households have about 1.2 ha of land with most households landless (WFP 2015). This has led to settlements on marginal land such as grazing areas, which has affected livestock keeping; the number of animals kept decreasing. The status of the nature of livelihoods as expressed in scholarly articles showed that in this district, agriculture is regarded as important although insufficient to provide enough (Gerhardt & Nemarundwe 2006; Scoones et al. 1996). A shift from maize production was also noted in some of the studies with farmers preferring small grain like sorghum and millet as these crops are more drought resistant (Mutopo 2014). Rural women in this area also dominate with a larger number of women than men; however, access to and control of resources remain a male-dominated area (Makura-Paradza 2010). These challenges that women face have led them to come up with coping strategies that cushion these challenges such as diversification of livelihoods and adoption of non-farm activities for income. The map (Figure 1) shows the case study area location in Zimbabwe.

## Breaking the cycle of gender discrimination against women

The sustainable development goals (SDGs) pushed for gender equality and the empowerment of women in all areas to achieve sustainable development (Hak et al. 2016; Sachs 2012). Rural women spend most of their time working on unpaid chores and lack opportunities such as access to resources, to make their time a lot more productive. The larger number of people in the rural areas are women; according to Zimstats (2012), the number of women in Chivi District is 90 170, while that of men is 75 879. Of the 166 049 people in Chivi District, 161 757 people live in the rural area making up 97% of the population. In most cases, rural areas are behind in development and well-being of the larger number of people; this is because of poor or lack of access to resources such as new technologies and improved methods of doing things, and lack of capital to diversify and improve their livelihoods. This has made rural areas prone to poverty (Ogunlela & Mukhtar 2009). This is also increased by the fact that most parts of Zimbabwe are patriarchal societies that have socio-cultural and political notions in favour of men (Mutopo 2014). Patriarchy has led to the marginalisation of women in accessing productive resources, markets and services.

There is a wide recognition by scholars and other development players that in most countries, women in agriculture face much more challenges than their male counterparts (Mutopo 2014; Oberhauser et al. 2004; UNDAW & UNIFEM 2001; UNDAW & UNDESA 2008). This has led to a call for governments and institutions of development to include women in all developmental strategies, policies and legislation, so as to ensure that they reach their full capacity (FAO 2011). Sustainability of agricultural development calls for the closure of gender gaps; the argument is that this closure would bring about an increase in agricultural productivity, reduction of hunger and poverty as well as the promotion of economic growth (FAO 2011). This equality should also be incorporated even at household levels where women should be empowered to make major decisions and have access to resources and household income; this would be ideal because it would strengthen the position of women outside the home as well.

## Theoretical framework

This article is underpinned by the sustainable livelihoods framework (SLF), which helps to explain rural women’s livelihoods that target economic empowerment in semi-arid regions of Zimbabwe. The sustainable livelihoods framework is undeniably helpful in the understanding of rural livelihoods and their sustainability. This framework was used to understand in particular the nature of livelihoods of rural women in Chivi District. It is helpful to the understanding of the distribution of work and chores within households between men and women (Chirau et al. 2014). The framework is also helpful in the understanding of socio-cultural, economic and political factors at play and how they are restrictive or supportive in the sustainability of rural livelihoods, especially those of women (Makura-Paradza 2010). Although the approach emphasises the strengths of people, it largely encompasses issues supporting environmental, socio-economic and institutional stability.

The SLF focuses on five forms of capitals or assets important to the sustainability of livelihoods, which are human, social, physical, natural and financial upon which people build their livelihoods. The implementation of the SLF begins by looking at the available assets of an individual or households; these are the things owned and controlled by a family (Scoones 1998). These assets allow the people to survive, engage in markets and participate in various activities within a community. The framework has also become popular as a way of conceptualising the economic activities of disadvantaged people in communities. The argument is that livelihoods are all about assets, systems, structures, sources of subsistence, income and position in societies (Vercillo 2016). A livelihood is believed to be sustainable if it can survive various shocks and stresses that arise (Scoones 1998). The key issues to achieve sustainable livelihoods include access and control over productive resources, entrepreneur development work, climate change and adaptation and women’s economic empowerment (Bird & Shepherd 2003; Hoddinott 2006; Mutami & Chazovachii 2012; Shackleton et al. 2001).

The SLF has five major sections which are vulnerability context, livelihood assets, transforming processes and structures, livelihood strategies and livelihood outcomes (Scoones 1998). On vulnerability context, the framework dwells on the trends, seasonality, as well as shocks and stresses that have an impact on the decision made by people on livelihoods. The vulnerability context looks at the risks and challenges that rural women’s livelihoods are exposed to in Chivi District. These challenges include lack of access to resources, climate change, gender roles and lack of capital. The asset briefcase holds human capital that includes education, skills and labour capacity of people to pursue a certain activity (Scoones 1998). Social capital includes the status bearing of women in societies and the need to eradicate discriminatory notions, family and community support as a social capital helps to support the promotion of sustainable livelihoods. Natural capital is the access to natural resources which promote the livelihoods such as land. According to Mutopo (2011), rural women lack access to productive resources such as capital and land which hinder the viability of their livelihoods. Financial capital is related to access to credit and finance resources; rural people lack access to credit facilities because of lack of assets they can use as collateral; land ownership is communal and not by title deeds which make it difficult to build assets for credit (Mutopo 2014). Physical capital entails the infrastructure that is available to women which improve their access to water, health care, housing, communication and other social services that people draw from for sustainability of livelihoods (Vercillo 2016). The transforming structures are referred to as those institutions and organisations affecting the use of various capitals people have in order to pursue their livelihoods (Vercillo 2016). Rural women pursue these livelihoods to earn income and achieve households, family well-being, security and other productive things of life. Makura-Paradza (2010) argues that a household is said to have a sustainable livelihood by the realisation of outcomes such as increased household or family well-being, improved food security, reduced vulnerabilities as well as the improved sustainable use of resources.

## Materials and methods

This article sets to assess the various rural women’s livelihood strategies used by women to mitigate poverty and improve household income in Chivi District. In their quest to reduce poverty, women in Chivi District were affected by various challenges which act as obstacles to women’s economic empowerment. These have been elucidated below.

### Design

The primary purpose of this article was to identify, describe and analyse rural women’s livelihoods and how they impact on women’s economic empowerment in Chivi District of Zimbabwe. This article used a case study research design where reports from previous studies were explored in order to understand complex and specific phenomena based on the ‘multiplicity of perspectives which are rooted in a specific context’ (Ritchie & Lewis, 2010). This design is explanatory in nature and is regarded as a robust research method, specifically when a holistic in-depth investigation is required (Gulsecen & Kubat 2006; Johnson 2006). Creswell (2013) affirms that:

a case study design explores a real-life, contemporary bounded system (a case) or multiple bounded systems (cases) over time, through detailed, in depth data collection involving multiple sources of information… and reports a case description and case themes. (p. 97)

A case study design, as Gillham (2000) observes, enables an inquiry to answer the research questions of a study. The design seeks a variety of diverse evidence from the case setting which is the social environment. The role of a case study design becomes more prominent in issues related to poverty and unemployment; hence, it has been used in this article to explain the nature of rural women’s livelihoods for economic empowerment in Chivi District of Zimbabwe. The case study design enables the researcher to examine the sustainability of rural women’s livelihoods and assess if household income has been improved and poverty reduced among women.

### Sampling technique

The study adopted a purposive sampling technique as the researchers intended to interview the participants with the intimate knowledge of participation in rural women’s livelihoods in selected villages in Chivi District. The sample of this article consisted of 40 women who were selected from an estimated population of 160 women involved in rural livelihoods across many villages in Chivi District. Only 40 women were chosen as participants based on their involvement in various women’s livelihood projects such as agriculture and pottery among others. Two focus group discussions (FDGs) consisting of ten people in each group were used for this study, and 20 women involved in rural women’s livelihoods were interviewed to get in depth data on how rural women’s livelihoods transform their standards of living in the semi-arid district. FDGs were used to enable many rural women who engage in various livelihood strategies to participate in the study.

### Data analysis

This article employed semi-structured interviews and documents to collect data. Semi-structured interviews were used because they allowed the researchers to conduct a person-to-person interaction; hence, face-to-face conversation increases the response rate by allowing the use of pictures, gestures and words (Kumar 2011). The main objective of this article was to examine the effectiveness of rural women’s livelihood towards rescuing poverty and economically empowering women in Chivi District. Therefore, interviews and focus group discussions (FDGs)were transcribed verbatim and presented using themes emerging from the article’s objectives. Thematic analysis, therefore, was used for analysing the secondary data based on the research objectives of this study. Boyatzis (1998) explains that thematic analysis identifies analyses and reports patterns (themes) within data. It organises and describes data in detail and interprets various aspects of the research topic. Therefore, in this article, thematic analysis was fundamental for identifying relevant data on the rural women’s livelihoods in Chivi District; it interprets and analyses the livelihoods to see if they are sustainable enough to enhance women’s economic empowerment in the drought-ridden district.

## Results and discussion

### The lack of access to competitive markets

Women in semi-arid regions of Zimbabwe are the front runners of the entrepreneurial economy through various rural livelihood strategies such as pottery, cooperatives vending and agriculture among others. In Chivi District, rural women’s livelihoods practised are mainly irrigation schemes and pottery. These economic empowerment initiatives are constrained by a lack of access to competitive markets. As a marginalised district, Chivi District does not provide ready markets, and hence, distant markets are favourable although these women do not have adequate transport to ferry their goods for selling. Studies conducted by Ncube (2012) and Mutami and Chazovachii (2012) attest to these findings when they revealed that women in developing countries are facing unequal barriers in accessing and standing market competitions. Although the livelihoods are aimed at women’s economic empowerment, the unavailability of ready markets incapacitates women in Chivi District. For example, favourable markets are in remote areas such as Masvingo town some 60 km – 70 km away. Lack of transport is another hindrance to transporting products to markets in time hence discouraging women’s economic empowerment.

An in-depth interview with one participant revealed this challenge when the woman stated that:

‘I have established my skills in making pots, I make various pots for different purposes, in towns most people like the decorative pots that I design for their displays but the major problem is how to carry the pots in bulk to town for selling. The towns are very far from here and I do not have reliable transport considering the fragility of my items. This has made it very difficult to earn from pottery as I end up selling the pottery along the main road. When I grow vegetables in my garden I can only rely on other villagers to come and buy or send to nearby school to sell to teachers. If sales are poor I am forced to dry the vegetables which are very difficult to sell.’ (Female Villager Participant, in her 50s)

The World Bank (2007) argues that to achieve economic empowerment, women should be empowered to become competitive. This is imperative for closing the economic gap between men and women in business. Therefore, to achieve women’s economic empowerment in semi-arid regions, information dissemination on the availability of ready markets should be increased among rural women in Chivi to avoid trading their agricultural outputs in domestic markets at a lower price because of the lack of access to markets. Therefore, to increase the competitiveness of markets, women need to be educated and informed on how to use modern technologies where they can access other favourable and effective markets.

### The need to micro-insure rural women’s livelihoods

It is common knowledge that financing rural women’s livelihoods in semi-arid regions of Zimbabwe has been done by non-governmental organisations (NGOs) because of government failure to fund such projects. Non-governmental organisations lack adequate funding because of changing priorities in the donor countries and other harsh government measures that always discourage fundraising; hence, rural women’s livelihoods often suffer from lack of funding. Findings from key informant interviews revealed that in Chivi District rural women’s livelihoods are mostly uninsured which results in their failure due to economic trends and lack of financial backup. Talking to these women, it emerged that most of these women did not know about insurance and its purpose. One woman said:

‘I have no idea what insurance is all about. In the villages, if you lose property due to fire you can only rely on neighbours’ generosity to help you. In cases of thefts and crop destruction, we used to have a community field where everyone would go and work and the produce will be kept by the chiefs to give out to the poor. However, people nowadays kept the produce for themselves.’ (Female Community Participant, in her 40s)

Most rural women’s livelihoods in Chivi District targeting women’s economic empowerment are not insured from risks such as fires, floods, thefts and droughts. In some instances where drought affected Chivi District, women suffered disproportionally, as they are the ones championing rural livelihoods for improving household income in many households (Mutami & Chazovachii 2012; Mutopo 2014). The socio-economic needs of women are affected as there is no insurance to cover for dry spells or other unintended consequences of climate change. This article argues that micro-insuring rural women’s livelihoods is a fundamental step in promoting women’s economic empowerment and improving the standards of living in the impoverished district. A study by Bajpai (2014) advocates for micro-insuring of rural women’s livelihoods, especially when they start their business which is tantamount to their sustainability. Rural women’s livelihoods in semi-arid regions often fail to be operational at the early stages; hence, micro-insuring is essential for growth and expansion. This has been revealed by a study conducted by Chazovachii and Chuma (2013) when it revealed that rural livelihoods are not micro-insured against any risk that may occur such as floods, fires, droughts and earthquakes. To this end, the lack of micro-insurance is detrimental to rural women’s livelihoods and economic empowerment in Chivi District.

### The need to increase access to credit facilities

Findings from FGDs in Ward 13 revealed that women in the Chivi District are hindered by limited access to credit facilities to finance rural women’s livelihoods. The reason stems from the fact that, as rural women, they do not have the collateral to convince the bank and other money lenders so that they extend lines of credit to them. Participants revealed that this has been a constraining factor for a long time as women clubs often fail to be sustainable because of lack of access to credit facilities.

One member of the focus group in Ward 13 stated that:

‘We do not have access to loans or money to support our small businesses as many of us are very poor and do not have assets to approach banks or other money lending institutions. Because of our poverty, we are afraid to borrow money since we do not have the collateral security required by the financial institution for them to recover their loans in the event we fail to pay back. This is a big challenge to us which disadvantage us to improve our household income and reduce poverty.’ (Female Village Participant, in her 30s)

From this assertion, lack of credit facilities hinders rural women’s livelihoods in Chivi; hence, economic empowerment is a challenge. In the agriculture sector, women lack enough subsidies to plant and grow their crops in time. This is attributed to the lack of access to credit facilities from the government; hence, rural women’s livelihoods are not viable because of cash shortages. The limited access to credit facilities negatively affects rural women’s livelihoods and discourages women’s economic empowerment. Studies by Banerjee, Karlan and Zinman (2015) and Banerjee (2013) show that microcredit is not a panacea to women’s economic empowerment. These results contradict the findings of this study that revealed that in Chivi District, the provision of credit facilities can help reduce poverty and economically empower women through rural women’s livelihoods. Empirical evidence from the studies revealed that micro-lenders charge micro-creditors high interests that have negative impacts on serial borrowers as they get trapped in debt. Findings of these studies point out further that success is not guaranteed by borrowing money from micro-lenders. The results of Banerjee et al (2015) concur with the findings of this study which show that economic empowerment through rural women’s livelihoods can be achieved in the event women are given access to credit facilities. Therefore, micro-crediting can be a strategy for the emancipation of women through resilient strategies; however, caution needs to be exercised for the funding from donor agencies or government may have regulations tied to them which can hinder the economic empowerment of women.

### The need to train women for economic empowerment

Bhavesh (2014) argues that skills training and education provide women with a basis for positive change and transformation in societies. Once women are empowered with skills, they can become innovative in their thoughts and understanding towards developing self and surrounding. In impoverished regions, the training of rural women can lead to economic empowerment as they are trained on how to effectively manage resources and tapping into the opportunities in order to create a value generation on a micro (household) level and a macro (societal level) level. Findings of this study have shown that rural women’s livelihoods are essential for job creation and income regeneration in Chivi District. Nevertheless, the sustainability of rural women’s livelihoods in Chivi District is being threatened by limited skills on how to effectively execute business decisions to improve household income. Although significant contributions made by agriculture as one of the main rural women’s livelihoods in Chivi were noticed, limited skills on how to improve on farming methods or how to cope in times of droughts and flooding. The lack of vocational schools to train and provide women with business management and entrepreneurship knowledge is a barrier in Chivi District to achieve women’s economic empowerment. One participant interviewed remarked that:

‘No organisation has provided training to us on how we can grow our businesses. Only in agriculture, the Agricultural extension officer visited once but we were not given enough education on how to grow cash crops which can increase our household income. Our limited knowledge is a barrier as we venture into less attractive livelihood programmes …’ (Female Community Participant, in her late 50s)

A holistic approach to enhance the agrarian and entrepreneurial education of women is paramount in Zimbabwe in order to achieve women’s economic empowerment. A study by Akhalwaya and Havnega (2012) revealed that limited education and training of women discourages women’s economic empowerment. It has been deduced from the findings of this study that education is fundamental to women as they can utilise it to advance rural women’s livelihoods and economic empowerment in Chivi District. Another study by Tornqvist and Schmitz (2009) attests to the arguments when it revealed that, vocational education for young women is vital for accelerating women’s economic empowerment in rural communities. Young, economically active women have the energy to drive economic change through sustainable rural livelihoods as they are better positioned to construct sound decisions. Education instils confidence in women which can be fundamental for socio-economic transformation and networking in rural women’s livelihoods.

### Mitigating the effects of climate change to enhance women’s empowerment

Respondents from the interviews revealed that climate change has serious effects in the Chivi District as it affects rural women’s livelihoods such as agriculture. According to the respondent, the intense global warming causes hot temperatures that negatively affect crop production. The women often do not obtain adequate information or early warnings that can be used as resilient strategies to cope in the face of climate change. In an FGD, one participant stated that:

‘The major problem is that we as women we do not know anything about climate change and how we can prevent it. When cyclones and heat waves occur our agricultural production is affected. Most families rely on past seasons yields for seeds (*mbeu*) and have to scrounge for money to buy fertilisers. It is quite sad because we have continuously lost our hard earned produce to erratic climatic conditions either the water will be too much and drown everything or the sun comes and burns everything.’ (Female, Village Participant)

Findings of this study revealed that climate change is affecting water and sanitation as well. This is because agriculture as a rural women’s livelihood depends on dammed water, so dry spells affect water levels to sustain agricultural production. Climate change negatively affects rural women’s livelihoods in Chivi as the majority of women are not aware of how risks emanating from climate change can be mitigated which often result in poor crop harvests in the agriculture sector. These findings are in conjunction with a study conducted by Anjani et al. (2013), which revealed that the lack of awareness on the causes and consequences of climate change is an obstacle to rural women as they are not aware of the dangers and adaptation mechanism on climate change. These findings show the significance of information on climate change among rural women. This article, therefore, advocates for early warning systems for rural women in semi-arid regions, so that they know which livelihood strategy to embark on depending on the weather or climate awareness information.

### Provision of modern technology to stimulate rural women’s livelihoods

The global technological inventions have engulfed the world of business and economic growth; hence, organisations and individuals need to keep abreast of such latest developments. The findings of the study revealed that in Chivi District, the majority of rural women are not well capacitated on how to use modern technological devices such as computers and smartphones to conduct extensive marketing for their products. The use of technology increases women’s productivity and decision-making power in their livelihood strategies. Findings of this study revealed further that women in Chivi are lagging behind in terms of how to use modern technological devices to increase their productivity and access to competitive markets. The limited skills and lack of awareness on how to effectively utilise the aforementioned technological devices are a barrier to women’s economic empowerment. One female participant lamented:

‘We do not have access to technology such as computers that can be useful for improving marketing and access to information on our livelihoods. We have made several requests to the government and stakeholders to assist us without any success. Anyhow, we need technical knowledge on how to use these modern machines.’ (Female Village Participant, early 30s)

Studies by WFP (2014) and World Bank (2014) recognised that when there are modern technological devices to improve agriculture, women suffer by using handmade tools which are not only inefficient but unproductive in an effort to reduce poverty levels. The article argues, therefore, that increased awareness of Information and Communication Technology services (ICTs) and their implementation can help bridge the socio-economic gap in Chivi District on the same note empowering women to sustain their livelihoods.

## Limitations of the study

The study was limited by the harsh geographical area of Chivi District as many of the rural women’s livelihoods were in remote areas that are inaccessible. Insufficient funding was a challenge to this study for the researcher to reach all rural livelihood strategies in the district. To mitigate these obstacles, the researchers purposively selected rural women’s livelihoods that were in selected accessible villages of Chivi District.

## Conclusion and recommendations

The study has shown that rural women’s livelihoods have the potential to empower women economically in semi-arid regions of Zimbabwe. Nevertheless, a myriad of factors affect rural women’s livelihoods and hinder economic empowerment. The article observes the severe lack of competitive markets that have compromised the profit margins of rural women. The abundance of rural livelihoods calls for ready markets; hence, their absence led rural women to look for local markets that are not profitable. The study ascertains that the lack of access to credit facilities is the main factor behind poverty among women in Chivi District. Presently, there are limited financial institutions that are willing to sponsor rural women to improve their livelihoods. The lack of training on how to use modern technological devices such as computers and internet is a setback as rural women are not exposed to external markets; hence, limited networking hinders economic empowerment. If empowered with necessary skills, women can self-sustain themselves through livelihood strategies in the process of increasing household income. The researchers concluded that rural women’s livelihoods in Chivi District can be successful when they are trained on how to cope with changes brought by climate change and have access to markets and credit facilities.

Based on the conclusions drawn from this study, rural women in the Chivi District and the Government of Zimbabwe can benefit from this study in the event they adopt the recommendations. As a way of increasing access to competitive markets, communication channels between rural women in Chivi should be enhanced through the use of modern technological devices such as cell phones, computers, televisions and radios. This helps in identifying ready markets where women can sell their products and encourage women to diversify their rural women livelihoods and tap into other resilient strategies to increase women’s economic empowerment. The study further recommends the Government of Zimbabwe to increase access to credit facilities. This can be done by setting up other agricultural banks apart from the Agricultural Bank (AGRIBANK) that target women from impoverished regions to access credit facilities as long as they have sound livelihood projects. Extending the line of credit to rural women to enable them to finance rural livelihood projects, such as irrigation agriculture, that requires extensive funding to be viable. Interested stakeholders can also assist with adequate funding towards micro-insuring rural women’s livelihoods to achieve women’s economic empowerment. The study further recommends that the government provide vocational schools in Chivi where women can be trained on skills to engage in agriculture among other rural women’s livelihoods. Education and training of women will enable them to network with other women associations which is imperative for information sharing on how women can improve household income in Chivi District through rural women’s livelihood strategies. In order to mitigate the effects of climate change to enhance women empowerment, the government can conduct early warning systems through the meteorological department that are imperative to warn women in semi-arid regions who engage in agriculture on how they should cope in times of risks such as floods or intense heat conditions. Further quantitative and qualitative studies can be conducted to assess the numbers and experiences of rural women’s livelihoods that are affected by climate change and how the government and stakeholders can intervene to help them.
